# Feature Selection and Machine Learning Approaches in Prediction of Current E-Cigarette Use Among U.S. Adults in 2022

**DOI:** 10.3390/ijerph21111474

**Published:** 2024-11-06

**Authors:** Wei Fang, Ying Liu, Chun Xu, Xingguang Luo, Kesheng Wang

**Affiliations:** 1 West Virginia Clinical and Translational Science Institute, Morgantown, WV 26506, USA; fehbdoc@gmail.com; 2 Department of Biostatistics and Epidemiology, College of Public Health, East Tennessee State University, Johnson City, TN 37614, USA; liuy09@etsu.edu; 3 Department of Health and Biomedical Sciences, College of Health Professions, University of Texas Rio Grande Valley, Brownsville, TX 78520, USA; chun.xu@utrgv.edu; 4 Department of Psychiatry, Yale University School of Medicine, New Haven, CT 06516, USA; xingguang.luo@yale.edu; 5 Department of Biobehavioral Health & Nursing Science, College of Nursing, University of South Carolina, Columbia, SC 29208, USA

**Keywords:** e-cigarette use, beliefs, binge drinking, PHQ4, feature selection, machine learning

## Abstract

Feature selection is essentially the process of picking informative and relevant features from a larger collection of features. Few studies have focused on predictors for current e-cigarette use among U.S. adults using feature selection and machine learning (ML) approaches. This study aimed to perform feature selection and develop ML approaches in prediction of current e-cigarette use using the 2022 Health Information National Trends Survey (HINTS 6). The Boruta algorithm and the least absolute shrinkage and selection operator (LASSO) were used to perform feature selection of 71 variables. The random oversampling example (ROSE) method was utilized to deal with imbalance data. Five ML tools including support vector machines (SVMs), logistic regression (LR), random forest (RF), gradient boosting machine (GBM), and extreme gradient boosting (XGBoost) were applied to develop ML models. The overall prevalence of current e-cigarette use was 4.3%. Using the overlapped 15 variables selected by Boruta and LASSO, the RF algorithm provided the best classifier with an accuracy of 0.992, sensitivity of 0.985, F1 score of 0.991, and AUC of 0.999. Weighted logistic regression further confirmed that age, education level, smoking status, belief in the harm of e-cigarette use, binge drinking, belief in alcohol increasing cancer, and the Patient Health Questionnaire-4 (PHQ4) score were associated with e-cigarette use. This study confirmed the strength of ML techniques in survey data, and the findings will guide inquiry into behaviors and mentalities of substance users.

## 1. Introduction

Electronic cigarettes (e-cigarettes) are also known as “vapes”, “e-hookahs”, or “electronic nicotine delivery systems (ENDS) [1]. Although e-cigarettes are commonly perceived as less harmful than cigarette smoking, it is important to note that most e-cigarettes contain nicotine, and their aerosol can contain substances harmful to the body [2,3,4]. Recent studies have shown that e-cigarettes may be associated with an increased risk of heart conditions [3,5], cancer outcomes [6,7], lung problems [8,9,10], and mental health problems and addiction [3,11]. A previous study has shown that e-cigarette use in the United States (U.S.) has increased rapidly over the past ten years [12]. For example, 3.7% of U.S. adults currently used e-cigarettes in 2020 [13], while 3.3% of middle school students and 14.1% of high school students used e-cigarettes in the past 30 days [14]. In addition, there are gender [15] and ethnic differences [16,17] in e-cigarette use.

Understanding the drivers of e-cigarette use holds significant value for theory and practice. Sociodemographic factors such as young adulthood, male gender, lower levels of education and income, and poor health have been demonstrated to be associated with e-cigarette use [11,18,19]. Furthermore, one previous review study reported that older age, male gender, conventional smoking, peer influence, daily smoking, and heavier smoking are the most common characteristics of adolescent e-cigarette users [20]. Another study reported that age, race/ethnicity, alcohol use, and depression, as well as novel factors associated with e-cigarette use, such as disabilities, obesity, history of diabetes, and history of arthritis, were identified using the Behavioral Risk Factor Surveillance System (BRFSS) [21]. One recent review study found that age, gender, cost of e-cigarettes, use of cigarettes, polysubstance use, and e-liquid nicotine concentration were associated with the escalation of e-cigarette use [22]. However, among all these findings, it appears that there is a lack of a strong association between sociodemographic factors and e-cigarette use [23], suggesting that e-cigarette use may vary between and within sociodemographic groups. Furthermore, the aforementioned research is mainly based upon regression analysis. Nevertheless, as e-cigarette use is a complex phenomenon, there may be a multitude of predictors at play behind the scenes, among which complex relationships such as high-order interactions might exist when regression analysis is not able to account for a great number of predictors and it is barely feasible to pre-specify complex relationships in a model.

Recently, with the rapid development of computer technology, a variety of machine learning (ML) methods such as logistic regression (LR), random forest (RF), support vector machine (SVM), gradient boosting machine (GBM), and extreme gradient boosting (XGBoost) models, capable of accounting for a large number of predictors and complex relationships, have been proposed to predict e-cigarette use or cessation [12,24,25,26,27,28,29,30]. The comprehensive Health Information National Trends Survey (HINTS), which collects nationally representative data about the American general public’s knowledge of, attitudes toward, and use of cancer- and health-related information, has been used to address e-cigarette-related issues [6,16,31,32,33,34,35,36,37]; however, no study has focused on using ML tools to predict e-cigarette use using the HINTS data. Furthermore, while it appears to make more sense to employ ML methods to predict e-cigarette use, identifying the most relevant factors is challenging. In particular, various sociodemographic factors, chronic conditions, alcohol, tobacco use, and knowledge and awareness of tobacco and health may be associated with e-cigarette use. However, the results are inconsistent, especially as some of these factors may be partially correlated. Thus, efficiently and accurately identifying factors that are most relevant to e-cigarette use from a plethora of cross-disciplinary candidates is crucial. As developing ML systems requires critical consideration of the specific features used in the analysis, feature selection is an important initial step. Feature selection, as a preprocessing stage, is essentially the process of picking some informative and relevant features from a larger collection of features to facilitate better characterization of patterns of multiple classes [38,39]. Several feature selection methods have been used in ML such as the least absolute shrinkage and selection operator (LASSO) [12,21,25,40] and the Boruta algorithm [21,40].

The present study aimed to perform feature selection and develop ML approaches in the prediction of current e-cigarette use using the 2022 HINTS data. The main contributions of this study are as follows:

1. The random oversampling example (ROSE) method [41], due to its simplicity and ease of implementation, was utilized to deal with imbalance data of two classes of current e-cigarette use (yes, no).
2. We used two feature selection methods (Boruta and LASSO) and identified the common features from both methods for further development of ML models.
3. We compared five ML tools (LR, SVMs, RF, GBM and XGBoost) to develop an ML model to predict current e-cigarette use. We used 10-fold cross-validation and tested multiple parameters for each algorithm using a grid search for optimal performance.
4. We applied a weighted logistic regression model to validate the independent variables with current e-cigarette use.

## 2. Materials and Methods

### 2.1. Sample

The sample selected from the 2022 Health Information National Trends Survey (HINTS 6) included 6252 respondents. Data collection for HINTS 6 started on 7 March 2022 and concluded on 8 November 2022. The overall household response rate using the next-birthday method [42] was 28.1%. There was an Institutional Review Board exemption for the present study since we conducted secondary data analysis using a publicly accessible database.

### 2.2. Outcome Variable

Current e-cigarette use was defined by the following two questions “Have you ever used an e-cigarette, even one or two times?” and “Do you now use an e-cigarette every day, some days, or not at all?”. Subjects were considered to have current e-cigarette use if they answered “Yes” to both questions.

### 2.3. Data Processing of Predictors

Demographic characteristics included gender, age group (18–34 years, 35–49 years, 50–64 years, 65 years or older), race, marital status (married/living together, widowed/divorced/separated, and never married), education, work full time (yes, no), income, and health insurance (yes, no). Race was recoded as Hispanic, non-Hispanic White, non-Hispanic Black or African American (AA), non-Hispanic Asian, and other. Education had four categories (less than high school, some college, bachelor’s degree, and post-baccalaureate degree). The four categories of annual income were <USD 19,999, USD 20,000–49,999, USD 50,000–74,999, and USD 75,000+.

**Figure 1:**
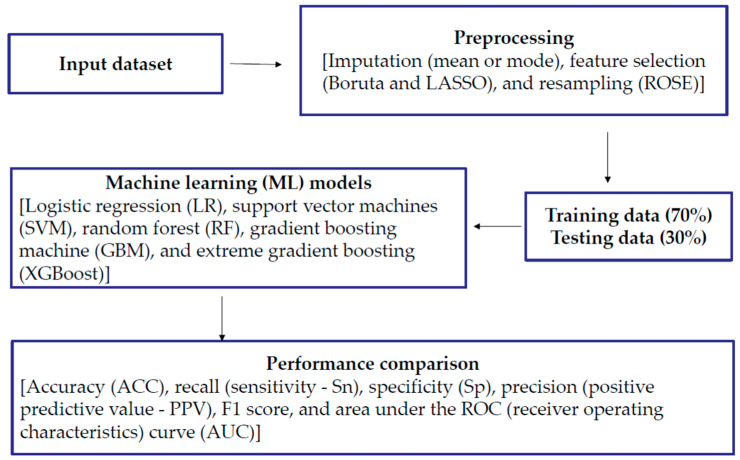
Overview of Data Curation and Machine Learning (ML) Workflow.

A total of 91 variables (including demographic factors and variables in alcohol and tobacco use, cancer information seeking, healthcare, chronic diseases, beliefs about cancer, health and nutrition, etc.) were included in the initial analysis. Variables with a missing value rate greater than 15% were removed based on previous simulation studies or real data analyses [25,28,43,44]. Individuals with missing values in outcome and nominal variables such as gender and race were removed, and there were then 5912 individuals left. Nominal variables were generated for dummy variables. Categorical variables with more than three levels were generated for dummy variables. Other variables were binary or ordinal or continuous. Then, missing values in continuous variables were imputed using the mean, and missing values in binary or ordinal variables were imputed by the mode. Figure 1 shows an overview of the data curation and ML process. A full list of the 71 remaining prediction variables is listed in Supplementary Table S1.

### 2.4. Feature Selection Methods and Resampling

Boruta [45] and LASSO [46] were used to select variables associated with the binary outcome. The Boruta algorithm in R (Version 4.4.2, R Core Team, Vienna, Austria), specifically the “Boruta” package, was used to automatically perform feature selection on a dataset using a random forest (RF) classifier [45]. The LASSO method in the R package “glmnet”, was also used to perform feature selection [46], which regularizes model parameter λ by shrinking the regression coefficients, reducing some of them to zero. The feature selection phase occurs after the shrinkage, where every non-zero value is selected as a parameter in the model. Considering the potential imbalanced data of two classes of current e-cigarette use (yes, no), the random oversampling example (ROSE) method was utilized. The method = “both” was selected, where both the minority class is oversampled with replacement and the majority class is under sampled without replacement [41].

### 2.5. Machine Learning Methods

Five ML algorithms were used including LR, SVM, RF, GBM, and XGBoost. The “caret” package in R was used for LR, SVM, RF, GBM, and XGBoost [47]. We used 10-fold cross-validation and tested multiple parameters for each algorithm using a grid search for optimal performance.

LR is a model of association between a dependent variable and independent variables when the dependent variable is binary; in this study, it is current e-cigarette use (Yes or No). We implemented the “glmnet” method in the “caret” package. In the grid search, we set alpha = 0:1 and lambda = seq (0.001, 1, length = 10).

SVM is a method of computing hyperplanes that optimally separate data belonging to two classes. In addition to linear classification, SVM also enables nonlinear classification using kernel tricks. The SVM algorithm includes linear kernel, radial kernel, and polynomial kernel [48]. In the grid search, for linear kernel, we set C = c(0.01, 0.1, 0.2, 0.5, 1, 2); for radial kernel, we set sigma = c(0.05, 0.25, 0.5, 1, 2) and C = c(0.05, 0.25, 0.5, 1, 2); and for polynomial kernel, we set C = c(0.05, 0.25, 0.5), degree = c(1, 2, 3), and scale = c(0.5, 1, 2).

The RF, which uses multiple decision trees, has been frequently used for classification. The RF algorithm randomly selects a subset of variables and constructs many decision trees [49,50]. In the grid search, we set mtry = c(1:15) and ntree = 300, where the mtry parameter refers to the number of variables used in each random tree, and ntree refers to the number of trees that the forest contains.

The GBM is an ensemble model where many weak classification tree models are converted into one single strong model to produce prediction [46]. The GBM is considered one of the most powerful boosting algorithms. In the grid search, we set interaction.depth = c(1, 5, 9), n.trees = (1:30) × 50, shrinkage = 0.1, and n.minobsinnode = 20, where the n.tress parameter refers to the number of trees, shrinkage is considered as a learning rate, and n.minobsinnode refers to the minimum number of observations in trees’ terminal nodes.

XGBoost [51] is a supervised machine learning method for regression and classification tasks like the RF classifier. However, it uses the CART (Classification and Regression Tree) and trains the trees serially and interactionally rather than in parallel and independently. In the grid search, we set the n_rounds = c(200, 300), max_depth = c(6, 10, 20), colsample_bytree = c(0.5, 1.0), eta = c(0.1, 0.3), gamma = c(0, 0.5), min_child_weight = c(1, 2), and subsample = c(0.75, 1.0), where n_rounds refer to the number of rounds for boosting, max_depth refers to the maximum depth of a tree, colsample_bytree refers to the subsample ratio of columns when constructing each tree, min_child_weight refers to the minimum sum of instance weight (hessian) needed in a child, and subsample refers to the subsample ratio of the training instances.

### 2.6. Performance of Machine Learning

The performance of ML methods was evaluated by measuring the accuracy (ACC), recall (sensitivity—Sn), specificity (Sp), precision (positive predictive value—PPV), F1 score, and area under the ROC (receiver operating characteristics) curve (AUC). The R packages used included “caret”, “kernlab”, and “ROCR”. The confusion matrix is illustrated in Table 1.

These measures are defined as follows:

(1) $$\mathrm{Accuracy}=\frac{\mathrm{TP}\;+\;\mathrm{TN}}{\mathrm{TP}\;+\;\mathrm{TN}\;+\;\mathrm{FP}\;+\;\mathrm{FN}}$$

(2) $$\mathrm{Recall}=\frac{\mathrm{TP}}{\mathrm{TP}\;+\;\mathrm{FN}}$$

(3) $$\mathrm{Specificity}=\frac{\mathrm{TN}}{\mathrm{TN}\;+\;\mathrm{FP}}$$

(4) $$\mathrm{Precision}=\frac{\mathrm{TP}}{\mathrm{TP}\;+\;\mathrm{FP}}$$

(5) $$F1\;\mathrm{Score}=2*\frac{\mathrm{Precision}*\mathrm{Recall}}{\mathrm{Precision}\;+\;\mathrm{Recall}}$$

Accuracy (ACC) is the ratio of correctly classified observations to the total number of observations.

Recall (Sensitivity—Sn) is the ratio of correctly predicted positive observations to all observations in the actual class—Yes.

Specificity (Sp)—the ratio of correctly predicted negative observations to all observations in the actual class—No.

Precision (positive predictive value—PPV) is the ratio of correctly predicted positive observations to the total predicted positive observations.

F1-Score is a harmonic mean that combines both recall and precision.

The AUC is the measure of the ability of a classifier to distinguish between classes and is served as a summary of the receiver operating characteristic (ROC) curve.

### 2.7. Statistical Analysis

The SURVEYFREQ procedure was used to weight and estimate population proportions of e-cigarette use across demographic variables. Weighted univariate and multiple logistic regression analyses using the SURVEYLOGISTIC procedure were performed to estimate the unadjusted and adjusted odds ratios (ORs and aORs) and 95% confidence intervals (CIs) for the associations of potential factors with e-cigarette use. All statistical analyses were performed using SAS statistical software, version 9.4 (SAS Institute, Cary, NC, USA).

## 3. Results

### 3.1. Prevalence of Current E-Cigarette Use

Among 5912 adult respondents, 191 reported being current e-cigarette users (Table 2). The overall prevalence was 4.3% (3.9% for males and 4.7% for females). The prevalence decreased with age (7.0%, 2.5%, 0.9% and 0.5% for age groups 18–34, 35–49, 50–64, and 65+, respectively).

### 3.2. Feature Selection and Resampling

**Figure 2:**
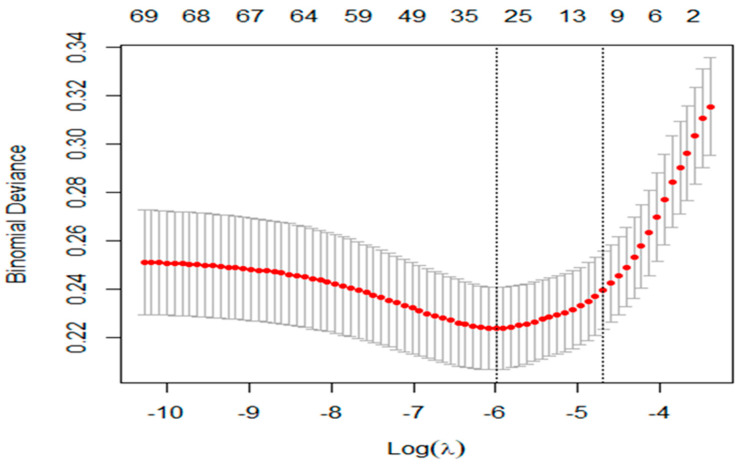
The LASSO software selected 30 variables on optimal parameter ln(λ) = −5.99.

**Figure 3:**
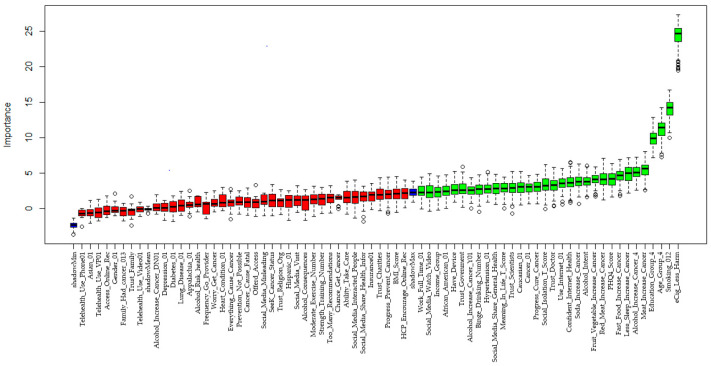
Variable importance plot in feature selection using Boruta algorithm. There are three regions highlighted by colors red, blue and green described in the legend. Boruta creates three areas—discard (red), speculative (blue) and keep (green)—to help identify the important features.

**Figure 4:**
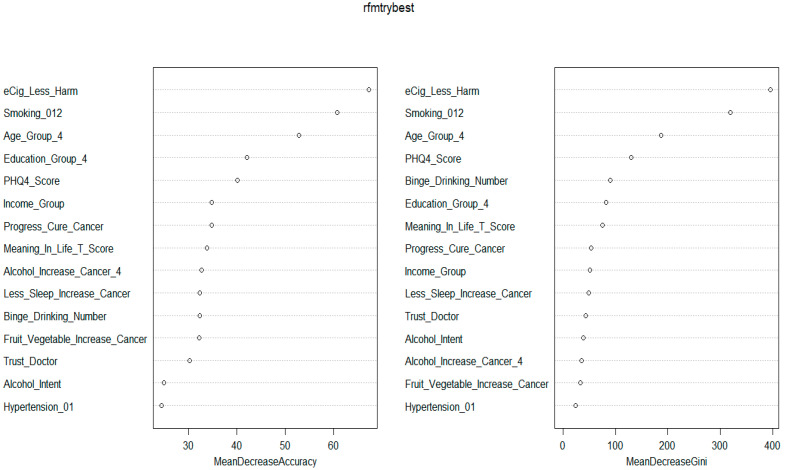
Plot of mean decrease accuracy (**left panel**) and mean decrease gini (**right panel**) values using random forest algorithm.

The LASSO method selected 30 features based on the optimal parameter ln(λ) = −5.99 (Figure 2 and Supplementary Table S1). Boruta selected 32 variables (Figure 3 and Supplementary Table S1). Boruta and LASSO selected 15 identical variables (Supplementary Table S1), which were used for developing ML tools. Figure 4 illustrates how the 15 selected variables performed via the RF algorithm. Based on the mean decrease accuracy, the top seven variables were belief in harm of e-cigarette use, smoking status, age, education level, belief in alcohol increasing cancer, belief in less sleep increasing cancer, and PHQ4 score (Figure 4 and Supplementary Table S1). PHQ4 score was used to assess the degree of depression severity via a questionnaire.

The HINTS 2022 has fewer current e-cigarette users (n = 191) than non-users (n = 5721). In order to generate balanced data, we used the “both” resampling method in ROSE, resulting in 2614 current e-cigarette users and 2653 non-users for further development of ML tools.

### 3.3. Machine Learning Performance

**Figure 5:**
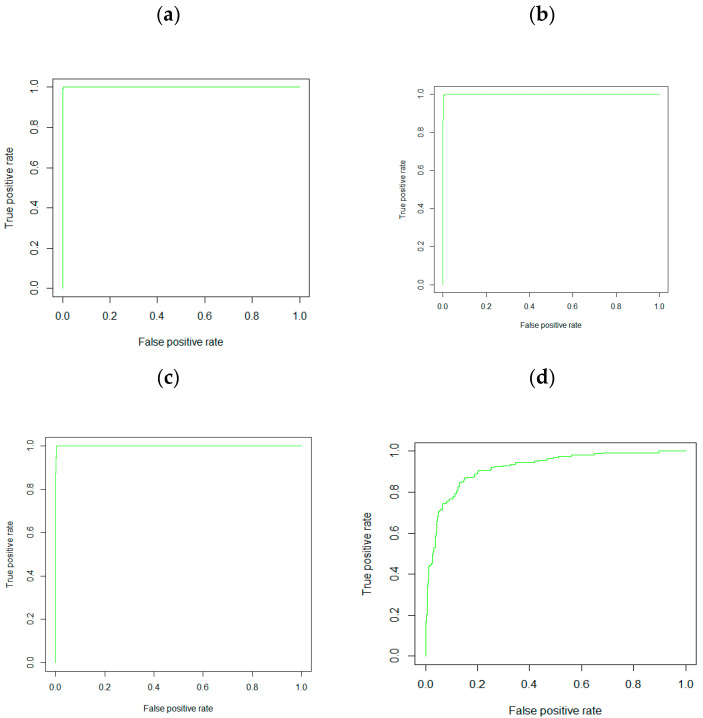
AUC curves in the validation data. (**a**) random forest, (**b**) XGBoost, (**c**) GBM, (**d**) logistic regression.

The performance statistics from the five ML tools are listed in Table 3. The RF, based on the 15 variables, provided the best classifier with the highest accuracy of 0.992, sensitivity of 0.985, F1 score of 0.991, and AUC of 0.999. The second-best model was GBM, which showed an accuracy of 0.989, sensitivity of 0.977, F1 score of 0.988, and AUC of 0.996. The AUC curves in the validation data for RF, XGBoost, GBM and LR models are illustrated in Figure 5.

### 3.4. Logistic Regression Analysis

Table 4 lists the results of the univariate and multiple logistic regression analyses of potential factors with e-cigarette use. Bivariate logistic regression analyses revealed that 8 of the 15 selected variables were associated with e-cigarette use. The multiple logistic regression analyses further revealed that binge drinking number, current and former smoking, and belief in harm of e-cigarette use were associated with increased odds (aOR (95% CI) = 1.44 (1.08–1.92), 11.29 (5.03–25.32), 6.99 (3.66–13.37), and 1.88 (1.38–2.56), respectively). Older age groups (35–49 and 50+ years old), bachelor’s degree, some college education, post-baccalaureate degree, and a belief in alcohol increasing cancer were associated with decreased odds (aOR (95% CI) = 0.30 (0.15–0.60), 0.11 (0.03–0.37), 0.50 (0.26–0.96), 0.25 (0.11–0.59), 0.13 (0.03–0.57), 0.72 (0.51–0.99), respectively).

## 4. Discussion

To the best of our knowledge, this study represents the first attempt to focus on feature selection and develop and validate ML tools to predict current e-cigarette use among U.S. adults using the latest HINTS 2022 data. The present study found that the overall prevalence of current e-cigarette use was 4.3% among U.S. adults in 2022, which is similar to 4.5% in 2019 [18] but a little higher than the reported 3.7% in 2020 [13]. Both Boruta and LASSO selected 15 identical variables. Based on the 15 selected variables, the RF yielded the most accurate classifier. Determined by the mean decrease accuracy in Boruta and RF, the top seven variables in predicting current e-cigarette use were belief in harm of e-cigarette use, smoking status, age, education level, belief in alcohol increasing cancer, belief in less sleep increasing cancer, and PHQ4 score. A weighted logistic regression model further confirmed that 6 of 15 variables including age, education level, smoking status, belief in harm of e-cigarette use, binge drinking, and belief in alcohol increasing cancer were significantly associated with current e-cigarette use.

Feature selection is a critical step in ML to reduce the dimensions of the feature space while revealing the most relevant features [52,53,54]. Previous studies have used several feature selection methods such as LASSO, Boruta, and RF [12,21,25,40]. For example, Boruta and LASSO were used to select variables associated with the current e-cigarette use, and both algorithms selected 26 identical variables [21]. Another study used a combined strategy of feature selection methods (such as LASSO and RF with ReliefF), identifying 40 predictor variables identified by both LASSO and RF [12]. One recent study used LASSO and selected 42 predictors to predict quitting smoking using e-cigarettes [29]. The present study applied Boruta and LASSO methods and selected 15 variables overlapped in both Boruta and LASSO (Supplementary Table S1). This overlap suggests that these features are robustly associated with the outcome and highlights the efficacy of combining feature selection methods to achieve reliable variable selection.

ML represents a powerful tool that could advance tobacco control research and policy decision-making [55]. ML has been used to predict e-cigarette use or cessation [12,24,25,26,27,28,29,30]. For example, RF has been used to identify important and unique distinguishing features between dual users and exclusive e-cigarette users, achieving 86.2% accuracy [27]. One study built prediction models for ever having used e-cigarettes or hookah by LASSO and RF with an accuracy of 0.637 and 0.734, respectively [12]. Another study developed a supervised ML prediction model using the penalized LR to discriminate between ENDS uses and non-uses, and the AUC for probability of current ENDS use ranged from 0.744 to 0.847 [26]. Furthermore, one Canadian study used the RF to predict daily vaping with a testing accuracy of 0.83, sensitivity of 0.85, and AUC of 0.90 [28]. A GBM model was used to classify vaping-assisted quitters and yielded a final GBM model with an AUC of 0.865, sensitivity of 0.85, and accuracy of 0.831 [29]. Recently, the XGBoost classifier was used for the prediction of ENDS initiation, and the maximum AUC for the XGBoost classifier was 0.82 [24]. A more recent study investigated the application of an eNose (electrochemical sensory array) device as a rapid and cost-effective screening tool to detect increasingly prevalent counterfeit electronic cigarettes, yielding the highest accuracy of 94.4% using an SVM algorithm [30]. Using the grid search, the present study compared five ML tools and found that the RF model was the best classifier with an accuracy of 0.992, sensitivity of 0.985, F1 score of 0.991, and AUC of 0.999 and outperformed LR, SVM, GBM, and XGBoost models (Table 3). Collectively, these findings suggest that RF models provide superior predictive performance for vaping-related outcomes, highlighting their robustness and potential in public health applications for tobacco use.

The variable importance measure based on Boruta and RF algorithms identified the belief in perceived lower harmfulness of e-cigarettes as the most important predictor associated with e-cigarette use, aligning with one recent study [31]. Several previous studies found that a reason for continuous use of e-cigarettes was mainly related to lower perceived harmfulness of e-cigarettes [56,57,58]. However, there is a lack of consensus in the literature about the association between the perceived health risks of e-cigarettes and their actual use among U.S. adults, an association that may depend on cigarette smoking status [31].

The second most important predictor was smoking status; the logistic regression confirmed that current and former smoking were associated with e-cigarette use, as previously reported [18,22,27,31,40]. For example, one recent study found that current cigarette smokers were more likely to engage in e-cigarette use than non-cigarette smokers were [31].

The present study further confirmed that young adults were more likely to engage in current e-cigarette use behavior [11,18,19,21,22,31,36,40], and a lower level of education has been found to have an association with e-cigarette use [11,16,18,19,21,36]. For example, one recent study found significantly elevated odds of e-cigarette use among young adults (18–34 years), whereas adults with the highest education levels had significantly lower odds of e-cigarette use [36].

This analysis shows that binge drinking is associated with e-cigarette use. One previous study found that alcohol consumption, including binge drinking and heavy drinking, had increased odds of e-cigarette use [21,24]. One review article also reported that polysubstance use was associated with escalation of e-cigarette use [22]. Furthermore, one Canadian study highlighted the importance of three substances, namely cannabis, alcohol and tobacco, to in the risk of ever-vaping use [28].

Additionally, our results added new findings to the literature on e-cigarette use, by identifying several new factors associated with e-cigarette use including belief in alcohol increasing cancer, belief in less sleep increasing cancer, and PHQ4 score. Interestingly, individuals who believe that alcohol and sleep deprivation increase cancer risk, as well as those with higher PHQ4 scores, are less likely to become e-cigarette users, based on the mean decrease in accuracy in Boruta and RF, belief in alcohol increasing cancer and belief in less sleep increasing cancer, and the PHQ4 score showed higher scores (>4.0). LASSO also confirmed the associations of these three variables with current e-cigarette use (Table S1). In weighted multiple logistic regression, high belief about alcohol_increase_cancer decreased odds of e-cigarette use (*p* = 0.0454), whereas both the bivariate and multiple logistic regression did not show a significant association of belief in less sleep increase cancer with e-cigarette use. PHQ4 score showed a highly significant association with e-cigarette use in the bivariate (*p* < 0.0001) but not multiple logistic regression, possibly revealing that PHQ4 has some correlation with other variables. The PHQ4 proved to be a reliable and valid screener for depression and anxiety. Previous studies have shown that compared with persons without the respective chronic health conditions, participants who reported mental health illnesses such as depression had increased odds of e-cigarette use [21,36]. Another study found that cancer survivors with lung disease or depression were more likely to use both cigarette and e-cigarette [7].

This study has boasted several notable strengths. Firstly, this study used the most recent HINTS (2022) data to examine the prevalence of current e-cigarette use. The HINTS data provide unparalleled insights into health information seeking, social media, and beliefs on alcohol and cancer. Secondly, we performed feature selection using LASSO and Boruta to identify common variables between these two methods. Thirdly, we compared five ML algorithms and found that the RF model has outstanding classification performance in predicting current e-cigarette use. Moreover, we used weighted logistic regression analysis to validate the results from ML techniques. However, our current analyses have limitations. Firstly, since the HINTS data are cross-sectional, we were only able to identify correlates; thus, the results cannot explain causal associations. Secondly, self-reported data on information seeking, social media, substance use, and beliefs regarding alcohol and cancer are prone to bias. Thirdly, the sample size of current e-cigarette users is relatively small. We used the random oversampling example (ROSE) method to deal with the imbalance data. Furthermore, the data are from 2022; the COVID-19 pandemic may have influenced the data collection and results.

## 5. Conclusions

The present study provided an update on the prevalence of current e-cigarette use among U.S. adults. Furthermore, we performed feature selection and compared five ML algorithms in the classification of current e-cigarette use and found that the RF offered the best performance. According to the mean decrease accuracy in Boruta and RF algorithms, the top seven predictors of current e-cigarette use were belief in harm of e-cigarette use, smoking status, age, education level, belief in alcohol increasing cancer, belief in less sleep increasing cancer, and PHQ4 score. In the multiple regression model, 6 of 15 selected variables were significantly associated with current e-cigarette use. These results provide valuable implications for future tobacco interventions aiming at maximizing the effectiveness of e-cigarettes as a potential cessation device. Our findings may benefit researchers, policymakers and healthcare providers by increasing public awareness, and supporting targeted e-cigarette education on e-cigarette use.

## Figures and Tables

**Table 1:** Confusion matrix.

**Confusion Matrix**		Predicted Value	
		Yes	No
Actual value	Yes	TP	FN
	No	FP	TN

TP is the number of true positives—the model correctly predicted a Yes outcome (the actual outcome was Yes), TN is the number of true negatives—the model correctly predicted a No outcome (the actual outcome was No), FP is the number of false positives—the model incorrectly predicted a Yes outcome (the actual outcome was No), and FN is the number of false negatives—the model incorrectly predicted a No outcome (the actual outcome was Yes).

**Table 2:** Prevalence of current e-cigarette use by demographic factor (%).

Variable	Total (N)	E-Cigarette	Prevalence (%) 95% CI	*p*-Value
Gender				
Male	2300	78	3.9 (2.0–5.9)	0.5292
Female	3516	109	4.7 (3.5–6.0)	
Age group				
18–34 years	2062	128	7.0 (4.9–9.1)	<0.0001
35–49 years	1694	47	2.5 (1.4–3.5)	
50–64 years	1326	14	0.9 (0.2–1.7)	
65+ years	830	2	0.5 (0.0–0.1)	
Race				
Non-Hispanic White	3193	114	5.0 (3.4–6.7)	0.2790
Non-Hispanic African American	885	17	2.4 (0.9–4.0)	
Hispanic	994	31	4.3 (2.1–6.4)	
Non-Hispanic Asian	288	4	2.0 (0.0–5.3)	
Other	184	15	4.4 (1.5–7.2)	
Education				
Less than High School	1441	66	6.3 (3.9–8.7)	0.002
Some College	1758	70	4.9 (2.9–6.9)	
Bachelor’s Degree	1609	46	2.5 (1.3–3.7)	
Post-Baccalaureate Degree	1104	9	1.2 (0.0–2.5)	
Income				
<19,999	1067	44	6.9 (3.4–10.3)	0.1933
20,000–49,999	1556	55	3.9 (2.2–5.5)	
50,000–74,999	996	32	3.3 (1.8–4.9)	
75,000+	2313	60	4..1 (2.0–6.2)	
Work Fulltime				
Yes	2766	109	4.5 (2.7–6.3)	0.7998
No	3049	79	4.2 (2.8–5.6)	
Insurance				
Yes	5403	163	4.3 (3.1–5.5)	0.7691
No	471	28	4.7 (2.2–7.2)	
Overall	5912	191	4.3 (3.2–5.4)	

Abbreviations: CI: Confidence interval. *p*-value calculated from χ^2^ test.

**Table 3:** Machine learning and comparison of performance.

Model	Variation	Accuracy	Sensitivity (Recall)	Specificity	Precision	F1-Score	AUC
SVM	Linear kernel	0.849	0.833	0.866	0.863	0.848	0.925
	RBF kernel	0.925	0.918	0.933	0.932	0.925	0.972
	Polynomial kernel	0.975	0.960	0.990	0.989	0.974	0.984
LR	Logistic regression	0.859	0.843	0.876	0.874	0.858	0.924
RF	Random forest	0.992	0.985	0.999	0.999	0.991	0.999
XGBoost	Extreme gradient boosting	0.988	0.976	0.999	0.999	0.987	0.996
GBM	Gradient boosting machine	0.989	0.977	0.999	0.999	0.988	0.996

Abbreviations: SVM: support vector machine; RBF: radial basis function; AUC: area under the ROC (receiver operating characteristics) curve.

**Table 4:** Univariate and multiple logistic regression analyses of current e-cigarette use.

Variable	OR (95% CI) ^1^	*p*-Value	aOR (95% CI) ^2^	*p*-Value
Age (ref = 18–34)				
35–49	0.34 (0.19–0.60)	0.0004	0.30 (0.15–0.60)	0.0009
50+	0.08 (0.03–0.21)	<0.0001	0.11 (0.03–0.37)	0.0007
Education (ref = less than high school)				
Some College	0.76 (0.43–1.34)	0.3311	0.50 (0.26–0.96)	0.0375
Bachelor’s Degree	0.38 (0.18–0.79)	0.0103	0.25 (0.11–0.59)	0.0020
Post-Baccalaureate Degree	0.18 (0.04–0.75)	0.0195	0.13 (0.03–0.57)	0.0074
Income (ref ≤ 19,999)				
20,000–49,999	0.53 (0.24–1.21)	0.1316	0.68 (0.23–2.04)	0.4804
50,000–74,999	0.46 (0.21–1.04)	0.0605	0.79 (0.25–2.48)	0.6828
75,000+	0.58 (0.25–1.33)	0.1934	0.80 (0.27–2.37)	0.6759
Trust doctor (1–4, 4 = a lot)	0.96 (0.66–1.41)	0.8454	0.98 (0.59–1.63)	0.9407
Alcohol_intent (1–4, 4 = Drink more alcohol)	1.29 (1.00–1.67)	0.0537	1.04 (0.75–1.44)	0.8072
Binge drinking number (1–5, 5 = 11 or more times)	1.87 (1.41–2.48)	<0.0001	1.44 (1.08–1.92)	0.0150
Alcohol_Increase_Cancer (1–4, 4 = a lot)	0.90 (0.68–1.18)	0.4226	0.72 (0.51–0.99)	0.0454
Smoking_Status (ref = never)				
Current	15.20 (7.40–31.19)	<0.001	11.29 (5.03–25.32)	<0.0001
Former	7.20 (4.25–12.21)	<0.001	6.99 (3.66–13.37)	<0.0001
eCigarette_Less Harm (1–7, 7 = much less harm)	2.01 (1.46–2.78)	<0.0001	1.88 (1.38–2.56)	0.0002
Hypertension (ref = no)	0.56 (0.32–0.98)	0.0440	0.81 (0.46–1.40)	0.4382
Less sleep increase Cancer (1–4, 4 = a lot)	1.30 (0.99–1.72)	0.0594	1.17 (0.76–1.80)	0.4677
Not Enough Fruit_Vegetable_Increase_Cancer (1–4, 4 = a lot)	1.18 (0.93–1.49)	0.1741	1.23 (0.85–1.78)	0.2729
Progress Cure Cancer (1–5, 5 = Do not know)	1.08 (0.93–1.26)	0.3274	1.01 (0.80–1.26)	0.9640
PHQ4 score	1.19 (1.12–1.27)	<0.0001	1.09 (0.98–1.20)	0.0996
Meaning_In_Life_T_Score	0.97 (0.95–0.99)	0.0011	1.01 (0.98–1.04)	0.6337

Abbreviations: OR ^1^ = crude odds ratio; aOR ^2^ = adjusted odds ratio; CI = confidence interval.

## Data Availability

The data that support the findings of this study are openly available in [https://hints.cancer.gov/data/default.aspx] at https://hints.cancer.gov/ (accessed on 16 December 2023).

## Supplementary Materials

The following supporting information can be downloaded at https://www.mdpi.com/article/10.3390/ijerph21111474/s1, Table S1: Feature selection based on LASSO and Boruta.

## References

[1] CDC (2024). About Electronic Cigarettes (E-Cigarettes).

[2] USDHHS (2016). E-Cigarette Use Among Youth and Young Adults: A Report of the Surgeon General.

[3] Coke L.A. (2020). Vaping and Use of E-Cigarette Products in Adolescents: A New Cardiopulmonary Crisis. J. Cardiovasc. Nurs..

[4] Huerta T.R., Walker D.M., Mullen D., Johnson T.J., Ford E.W. (2017). Trends in E-Cigarette Awareness and Perceived Harmfulness in the U.S. Am. J. Prev. Med..

[5] Ahmed N., Kalininskiy A., Gandhi H., Shin J.J. (2018). Spontaneous Coronary Artery Dissection in a Postpartum E-Cigarette Smoker. BMJ Case Rep..

[6] Bjurlin M.A., Basak R., Zambrano I., Schatz D., El Shahawy O., Sherman S., Matulewicz R.S. (2022). Perceptions of E-Cigarette Harm among Cancer Survivors: Findings from a Nationally Representative Survey. Cancer Epidemiol..

[7] Kim J., Keegan T.H. (2023). Characterizing Risky Alcohol Use, Cigarette Smoking, e-Cigarette Use, and Physical Inactivity among Cancer Survivors in the USA—A Cross-Sectional Study. J. Cancer Surviv..

[8] Alber J.M., Conover S., Marts E., Ganjooi K., Grossman S. (2021). Examining E-Cigarette Perspectives before and after the EVALI Peak in Cases. Addict. Behav..

[9] Bhatta D.N., Glantz S.A. (2020). Association of E-Cigarette Use With Respiratory Disease Among Adults: A Longitudinal Analysis. Am. J. Prev. Med..

[10] Gupta P.S., Kalagher K.M. (2021). Where There Is (No) Smoke, There Is Still Fire: A Review of Trends, Reasons for Use, Preferences and Harm Perceptions of Adolescent and Young Adult Electronic Cigarette Use. Curr. Pediatr. Rep..

[11] Obisesan O.H., Mirbolouk M., Osei A.D., Orimoloye O.A., Uddin S.M.I., Dzaye O., El Shahawy O., Al Rifai M., Bhatnagar A., Stokes A. (2019). Association Between E-Cigarette Use and Depression in the Behavioral Risk Factor Surveillance System, 2016-2017. JAMA Netw. Open.

[12] Choi J., Jung H.-T., Ferrell A., Woo S., Haddad L. (2021). Machine Learning-Based Nicotine Addiction Prediction Models for Youth E-Cigarette and Waterpipe (Hookah) Users. JCM.

[13] Cornelius M.E., Loretan C.G., Wang T.W., Jamal A., Homa D.M. (2022). Tobacco Product Use Among Adults—United States, 2020. MMWR Morb. Mortal. Wkly. Rep..

[14] Park-Lee E., Ren C., Cooper M., Cornelius M., Jamal A., Cullen K.A. (2022). Tobacco Product Use Among Middle and High School Students—United States, 2022. MMWR Morb. Mortal. Wkly. Rep..

[15] Yimsaard P., McNeill A., Yong H.-H., Cummings K.M., Chung-Hall J., Hawkins S.S., Quah A.C.K., Fong G.T., O’Connor R.J., Hitchman S.C. (2021). Gender Differences in Reasons for Using Electronic Cigarettes and Product Characteristics: Findings from the 2018 ITC Four Country Smoking and Vaping Survey. Nicotine Tob. Res..

[16] Assari S., Mistry R., Bazargan M. (2019). Race, Educational Attainment, and E-Cigarette Use. J. Med. Res. Innov..

[17] Barrington-Trimis J.L., Bello M.S., Liu F., Leventhal A.M., Kong G., Mayer M., Cruz T.B., Krishnan-Sarin S., McConnell R. (2019). Ethnic Differences in Patterns of Cigarette and E-Cigarette Use Over Time Among Adolescents. J. Adolesc. Health.

[18] Cornelius M.E., Wang T.W., Jamal A., Loretan C.G., Neff L.J. (2020). Tobacco Product Use Among Adults—United States, 2019. MMWR Morb. Mortal. Wkly. Rep..

[19] Owusu D., Huang J., Weaver S.R., Pechacek T.F., Ashley D.L., Nayak P., Eriksen M.P. (2019). Patterns and Trends of Dual Use of E-Cigarettes and Cigarettes among U.S. Adults, 2015–2018. Prev. Med. Rep..

[20] Gorukanti A., Delucchi K., Ling P., Fisher-Travis R., Halpern-Felsher B. (2017). Adolescents’ Attitudes towards e-Cigarette Ingredients, Safety, Addictive Properties, Social Norms, and Regulation. Prev. Med..

[21] Atuegwu N.C., Oncken C., Laubenbacher R.C., Perez M.F., Mortensen E.M. (2020). Factors Associated with E-Cigarette Use in U.S. Young Adult Never Smokers of Conventional Cigarettes: A Machine Learning Approach. Int. J. Environ. Res. Public Health.

[22] Short M., Cole A.G. (2021). Factors Associated with E-Cigarette Escalation among High School Students: A Review of the Literature. Int. J. Environ. Res. Public Health.

[23] Gaiha S.M., Rao P., Halpern-Felsher B. (2022). Sociodemographic Factors Associated with Adolescents’ and Young Adults’ Susceptibility, Use, and Intended Future Use of Different E-Cigarette Devices. Int. J. Environ. Res. Public Health.

[24] Atuegwu N.C., Mortensen E.M., Krishnan-Sarin S., Laubenbacher R.C., Litt M.D. (2023). Prospective Predictors of Electronic Nicotine Delivery System Initiation in Tobacco Naive Young Adults: A Machine Learning Approach. Prev. Med. Rep..

[25] Fu R., Shi J., Chaiton M., Leventhal A.M., Unger J.B., Barrington-Trimis J.L. (2022). A Machine Learning Approach to Identify Predictors of Frequent Vaping and Vulnerable Californian Youth Subgroups. Nicotine Tob. Res..

[26] Han D.-H., Lee S.H., Lee S., Seo D.-C. (2021). Identifying Emerging Predictors for Adolescent Electronic Nicotine Delivery Systems Use: A Machine Learning Analysis of the Population Assessment of Tobacco and Health Study. Prev. Med..

[27] Romijnders K.A.G.J., Pennings J.L.A., Van Osch L., De Vries H., Talhout R. (2019). A Combination of Factors Related to Smoking Behavior, Attractive Product Characteristics, and Socio-Cognitive Factors Are Important to Distinguish a Dual User from an Exclusive E-Cigarette User. Int. J. Environ. Res. Public Health.

[28] Shi J., Fu R., Hamilton H., Chaiton M. (2022). A Machine Learning Approach to Predict E-Cigarette Use and Dependence among Ontario Youth. Health Promot. Chronic Dis. Prev. Can..

[29] Fu R., Schwartz R., Mitsakakis N., Diemert L.M., O’Connor S., Cohen J.E. (2022). Predictors of Perceived Success in Quitting Smoking by Vaping: A Machine Learning Approach. PLoS ONE.

[30] Eng B., Dalby R.N. (2024). Applications of an Electrochemical Sensory Array Coupled with Chemometric Modeling for Electronic Cigarettes. Sensors.

[31] Adzrago D., Shi Y., Fujimoto K. (2023). Association between Perceived Health Risks of E-Cigarettes and Actual e-Cigarette Use, Based on Cigarette Smoking Status and Sexual and Gender Minority Status among U.S. Adults. J. Public Health.

[32] Cardona S., Calixte R., Rivera A., Islam J.Y., Vidot D.C., Camacho-Rivera M. (2021). Perceptions and Patterns of Cigarette and E-Cigarette Use among Hispanics: A Heterogeneity Analysis of the 2017–2019 Health Information National Trends Survey. Int. J. Environ. Res. Public Health.

[33] Cho B., Lee S., Pan Y., Sharma M., Holland K. (2023). Association of Cancer Information Seeking Behavior with Cigarette Smoking and E-Cigarette Use among U.S. Adults by Education Attainment Level: A Multi-Year Cross-Sectional Analysis from a Nationally Representative Sample in 2017–2020. Prev. Med..

[34] Ford E.W., Chan K.S., Parikh M., Lowe K.B., Huerta T.R. (2020). E-Cigarette and Hookah Adoption Patterns: Is the Harm Reduction Theory Just so Much Smoke?. Addict. Behav. Rep..

[35] Lewis-Thames M.W., Langston M.E., Fuzzell L., Khan S., Moore J.X., Han Y. (2020). Rural-Urban Differences e-Cigarette Ever Use, the Perception of Harm, and e-Cigarette Information Seeking Behaviors among U.S. Adults in a Nationally Representative Study. Prev. Med..

[36] Mamudu H.M., Adzrago D., Dada O., Odame E.A., Ahuja M., Awasthi M., Weierbach F.M., Williams F., Stewart D.W., Paul T.K. (2023). Examining Disparities in Current E-Cigarette Use among U.S. Adults before and after the WHO Declaration of the COVID-19 Pandemic in March 2020. Int. J. Environ. Res. Public Health.

[37] Zhang L., Qiu S.S., Ao S.H., Zhao X. (2024). Association between Health-Related Social Media Use and E-Cigarette Use among Current Cigarette Users: The Roles of Anti-Tobacco Messages and Harm Perception. BMC Public Health.

[38] Chen X., Kopsaftopoulos F., Wu Q., Ren H., Chang F.-K. (2018). Flight State Identification of a Self-Sensing Wing via an Improved Feature Selection Method and Machine Learning Approaches. Sensors.

[39] Raihan-Al-Masud M., Mondal M.R.H. (2020). Data-Driven Diagnosis of Spinal Abnormalities Using Feature Selection and Machine Learning Algorithms. PLoS ONE.

[40] Atuegwu N.C., Litt M.D., Krishnan-Sarin S., Laubenbacher R.C., Perez M.F., Mortensen E.M. (2021). E-Cigarette Use in Young Adult Never Cigarette Smokers with Disabilities: Results from the Behavioral Risk Factor Surveillance System Survey. Int. J. Environ. Res. Public Health.

[41] Lunardon N., Menardi G., Torelli N. (2014). ROSE: A Package for Binary Imbalanced Learning. R J..

[42] Salmon C.T., Nichols J.S. (1983). The next-birthday method of respondent selection. Public Opinion Quarterly. Public Opin. Q..

[43] Henry A.J., Hevelone N.D., Lipsitz S., Nguyen L.L. (2013). Comparative Methods for Handling Missing Data in Large Databases. J. Vasc. Surg..

[44] Fellinghauer C.S., Prodinger B., Tennant A. (2018). The Impact of Missing Values and Single Imputation upon Rasch Analysis Outcomes: A Simulation Study. J. Appl. Meas..

[45] Kursa M.B., Rudnicki W.R. (2010). Feature Selection with the Boruta Package. J. Stat. Soft..

[46] Friedman J., Hastie T., Tibshirani R. (2010). Regularization Paths for Generalized Linear Models via Coordinate Descent. J. Stat. Soft..

[47] Kuhn M. (2008). Building Predictive Models in *R* Using the Caret Package. J. Stat. Soft..

[48] Cortes C., Vapnik V. (1995). Support-Vector Networks. Mach. Learn..

[49] Chen W., Xie X., Wang J., Pradhan B., Hong H., Bui D.T., Duan Z., Ma J. (2017). A Comparative Study of Logistic Model Tree, Random Forest, and Classification and Regression Tree Models for Spatial Prediction of Landslide Susceptibility. CATENA.

[50] Kesler S.R., Rao A., Blayney D.W., Oakley-Girvan I.A., Karuturi M., Palesh O. (2017). Predicting Long-Term Cognitive Outcome Following Breast Cancer with Pre-Treatment Resting State fMRI and Random Forest Machine Learning. Front. Hum. Neurosci..

[51] Chen T., Guestrin C. (2016). XGBoost: A Scalable Tree Boosting System. Proceedings of the 22nd ACM SIGKDD International Conference on Knowledge Discovery and Data Mining.

[52] Awan S.E., Bennamoun M., Sohel F., Sanfilippo F.M., Chow B.J., Dwivedi G. (2019). Feature Selection and Transformation by Machine Learning Reduce Variable Numbers and Improve Prediction for Heart Failure Readmission or Death. PLoS ONE.

[53] Cai J., Luo J., Wang S., Yang S. (2018). Feature Selection in Machine Learning: A New Perspective. Neurocomputing.

[54] Cömert Z., Şengür A., Budak Ü., Kocamaz A.F. (2019). Prediction of Intrapartum Fetal Hypoxia Considering Feature Selection Algorithms and Machine Learning Models. Health Inf. Sci. Syst..

[55] Fu R., Kundu A., Mitsakakis N., Elton-Marshall T., Wang W., Hill S., Bondy S.J., Hamilton H., Selby P., Schwartz R. (2023). Machine Learning Applications in Tobacco Research: A Scoping Review. Tob. Control.

[56] Amrock S.M., Lee L., Weitzman M. (2016). Perceptions of E-Cigarettes and Noncigarette Tobacco Products Among US Youth. Pediatrics.

[57] Huang J., Feng B., Weaver S.R., Pechacek T.F., Slovic P., Eriksen M.P. (2019). Changing Perceptions of Harm of E-Cigarette vs Cigarette Use Among Adults in 2 US National Surveys From 2012 to 2017. JAMA Netw. Open.

[58] Manzione L.C., Shan L., Azagba S. (2020). Associations Between Risk Perceptions and Cigarette, E-Cigarette, and Dual-Product Use Among Canadian Adolescents. Tob. Use Insights.

